# Automatic view classification of contrast and non-contrast echocardiography

**DOI:** 10.3389/fcvm.2022.989091

**Published:** 2022-09-14

**Authors:** Ye Zhu, Junqiang Ma, Zisang Zhang, Yiwei Zhang, Shuangshuang Zhu, Manwei Liu, Ziming Zhang, Chun Wu, Xin Yang, Jun Cheng, Dong Ni, Mingxing Xie, Wufeng Xue, Li Zhang

**Affiliations:** ^1^Department of Ultrasound, Union Hospital, Tongji Medical College, Huazhong University of Science and Technology, Wuhan, China; ^2^Clinical Research Center for Medical Imaging in Hubei Province, Wuhan, China; ^3^Hubei Province Key Laboratory of Molecular Imaging, Wuhan, China; ^4^National-Regional Key Technology Engineering Laboratory for Medical Ultrasound, Guangdong Key Laboratory for Biomedical Measurements and Ultrasound Imaging, Shenzhen, China; ^5^School of Biomedical Engineering, Health Science Center, Shenzhen University and Medical Ultrasound Image Computing (MUSIC) Lab, Shenzhen, China; ^6^Media and Communication Lab (MC Lab), Electronics and Information Engineering Department, Huazhong University of Science and Technology, Wuhan, China

**Keywords:** echocardiography, contrast, view classification, convolutional neural network, artificial intelligence (AI)

## Abstract

**Background:**

Contrast and non-contrast echocardiography are crucial for cardiovascular diagnoses and treatments. Correct view classification is a foundational step for the analysis of cardiac structure and function. View classification from all sequences of a patient is laborious and depends heavily on the sonographer’s experience. In addition, the intra-view variability and the inter-view similarity increase the difficulty in identifying critical views in contrast and non-contrast echocardiography. This study aims to develop a deep residual convolutional neural network (CNN) to automatically identify multiple views of contrast and non-contrast echocardiography, including parasternal left ventricular short axis, apical two, three, and four-chamber views.

**Methods:**

The study retrospectively analyzed a cohort of 855 patients who had undergone left ventricular opacification at the Department of Ultrasound Medicine, Wuhan Union Medical College Hospital from 2013 to 2021, including 70.3% men and 29.7% women aged from 41 to 62 (median age, 53). All datasets were preprocessed to remove sensitive information and 10 frames with equivalent intervals were sampled from each of the original videos. The number of frames in the training, validation, and test datasets were, respectively, 19,370, 2,370, and 2,620 from 9 views, corresponding to 688, 84, and 83 patients. We presented the CNN model to classify echocardiographic views with an initial learning rate of 0.001, and a batch size of 4 for 30 epochs. The learning rate was decayed by a factor of 0.9 per epoch.

**Results:**

On the test dataset, the overall classification accuracy is 99.1 and 99.5% for contrast and non-contrast echocardiographic views. The average precision, recall, specificity, and F1 score are 96.9, 96.9, 100, and 96.9% for the 9 echocardiographic views.

**Conclusions:**

This study highlights the potential of CNN in the view classification of echocardiograms with and without contrast. It shows promise in improving the workflow of clinical analysis of echocardiography.

## Introduction

Transthoracic echocardiography is one of the most important non-invasive imaging techniques, with the advantages of non-radiation, easy bedside operation, and real-time evaluation (1). With changes in relative position between the transducer and the patient, sonographers obtain multiple views from different perspectives. In particular, the apical views and short axis views are most commonly used in routine clinical workflows (1, 2). Currently, view classification is the prerequisite for the post-processing and analysis of cardiac structure and function. However, it generally depends on the sonographer’s experience and is time-consuming especially for large datasets. It is a challenging task due to the inter-view similarity, intra-view variability and noise.

Earlier research adopted classical machine learning algorithms to classify echocardiograms with multiple views. They generally extracted features using the Histogram of Oriented Gradients (HOG) (3), Bag of Word (BoW) (4, 5), and classified echocardiography view using the Support Vector Machine (SVM) (3–7). Current studies have mainly focused on Convolutional neural networks (CNNs), which have brought about a series of breakthroughs for medical image analysis (8, 9). CNNs tend to recognize visual patterns from raw image pixels in an end-to-end learning process. The initial layers are used to observe local geometric structures (such as edges, blobs, etc.), whereas the neurons in the higher layers focus more on the global distribution of human organs. A large number of studies have confirmed the feasibility and accuracy of CNNs with various depth in echocardiographic view classification (10–13). For a closer look of the echocardiographic images, Madani et al. has used U-Net to extract the regions of interest, improving the signal-to-noise ratio. The precision and efficiency of the network were further promoted (14). Echocardiography has rich temporal domain information, while single CNNs only focuses on spatial location information. A study indicated that the dense optical flow technique represented temporal motion information, building two strands of CNNs with temporal-spatial information fusion and improving classification accuracy from 89.5% of single CNN to 92.1% of the fusion network (12). In addition, several studies optimized the algorithm based on CNNs, ensuring the classification accuracy and significantly improving efficiency. CNNs can be used to facilitate automatic multiplanar reformation and orientation guidance (15) and deploy on mobile devices for downstream analysis (16). CNNs simplify the image processing process, assist novices in identifying standard images, reduce observer variability, and improve analyzing efficiency.

Existing studies have mainly focused on two-dimensional grayscale or Doppler echocardiograms (3–7, 10–16). Most of them dealt with common cardiac views: apical two-chamber (A2C), apical three-chamber (A3C) and apical four-chamber (A4C), as well as the parasternal short-axis (PSAX). Although extensive studies have been carried out on conventional echocardiography, no single study focuses on the view classification of contrast echocardiography. Contrast echocardiography significantly enhances the boundaries of the left ventricular endocardium, which has great clinical significance in the quantification of cardiac function (2). In addition, contrast echocardiography effectively reduces missed diagnoses of apical hypertrophic cardiomyopathy (17), intracardiac thrombi, and non-compaction cardiomyopathy (18). However, the contrast agent fills the heart cavity, making the mitral valve ring obscure, which increases the difficulty of identifying the primary views. Therefore, this study sets out to evaluate the discriminative capability of CNNs in identifying the PSAX, A2C, A3C, A4C views from non-contrast or contrast echocardiographic videos.

## Materials and methods

### Study design

All datasets were collected from 855 patients who underwent left ventricular opacification at the Department of Ultrasound Medicine, Wuhan Union Medical College Hospital from 2013 to 2021. This study was approved by the Ethics Committee of Tongji Medical College, Huazhong University of Science and Technology, Wuhan, China. In the study population, 70.3% are male and 29.7% are female, aging from 41 to 62 with a median age of 53. Indications of left ventricular opacification in routine clinical practice are shown in Table 1. Each sample consisted of data from the echocardiographic examination of a patient, including M-mode, two-dimensional, three-dimensional, Doppler and other still images or videos. The echocardiograms were mainly acquired with GE Vivid E9, Philips iE33, IE Elite, EPIQ 7C, and EPIQ 5.

**TABLE 1:** Baseline characteristics.

Variable	All (*N* = 855)	Training (*N* = 688)	Validation (*N* = 84)	Testing (*N* = 83)
**Demographics**				
Age (years)	53 (41, 62)	53 (40, 62)	53 (42, 62)	51 (43, 63)
Sex (male)	601 (70.3%)	476 (69.2%)	65 (77.4%)	59 (71.1%)
**Indication**				
Myocardial hypertrophy	360 (42.0%)	285 (41.4%)	37 (44.0%)	38 (45.8%)
NCM	85 (9.9%)	61 (8.9%)	11 (13.1%)	13 (15.7%)
DCM	17 (2.0%)	12 (1.7%)	3 (3.6%)	2 (2.4%)
NCM & DCM	32 (4.0%)	29 (4.0%)	2 (2.4%)	1 (1.2%)
RWMA	118 (13.8%)	98 (14.2%)	13 (11.9%)	7 (8.4%)
Others	243 (28.4%)	203 (29.5%)	18 (21.4%)	22 (26.5%)

Data are expressed as median (interquartile range) or number (%). NCM, non-compaction of ventricular myocardium; DCM, dilated cardiomyopathy; RWMA, regional wall motion abnormality; Others, other conditions that required contrast echocardiography.

### Data preprocessing

The echocardiograms were stored in DICOM format. This study mainly analyzed two-dimensional grayscale videos (contrast and non-contrast echocardiography). All videos are anonymized.

The PSAX and apical views play an essential role in the diagnosis and treatment of cardiovascular diseases. The PSAX view focuses on mitral valve and left ventricular wall motion. The A2C, A3C, and A4C views are mainly used to assess cardiac structure and function comprehensively. Moreover, it is of incremental value for detecting apical abnormalities in contrast echocardiography. Thus, the dataset was divided into nine categories, including the above-mentioned views of contrast and non-contrast echocardiograms, and an additional class including all the rest views (parasternal left ventricular long axis, pulmonary artery long axis, and major artery short axis views, etc.). All views included diverse image quality and were reviewed independently by two experts. Low-quality and inefficient videos were excluded (contrast echocardiography with underfilling or echocardiography with the incompletion of heart chambers).

The dataset consisted of video clips from the 10 temporally sampled frames with equivalent intervals from each of the original sequences. Considering the different types of equipment for image acquisition, all clips were downsampled to 256 × 256 pixels by linear interpolation and the intensity is normalized into [0, 1]. Quality control is carried out by random sampling the preprocessed clips, to ensure that the dataset did not contain sensitive information. To maintain sample independence, all samples were randomly split into training, validation and test datasets in an approximate 8:1:1 ratio. The training dataset was used for model development. The validation dataset was used for tuning model parameters, and the testing dataset was used for evaluating the performance of the final model. The training, validation, and test datasets included 688, 84, and 83 studies, respectively (corresponding to 1,937, 237, and 262 clips from 9 views). The datasets are derived from real world data. The distribution of each dataset is shown in Table 2.

**TABLE 2:** Distribution of the clip number in the dataset.

Class	Training	Validation	Testing	Total
2DE.A2C	222 (2,220)	28 (280)	28 (280)	278 (2,780)
2DE.A3C	233 (2,330)	31 (310)	30 (300)	294 (2,940)
2DE.A4C	219 (2,190)	25 (250)	30 (300)	274 (2,740)
2DE.PSAX	226 (2,260)	29 (290)	31 (310)	286 (2,860)
C2DE.A2C	224 (2,240)	29 (290)	30 (300)	283 (2,830)
C2DE.A3C	182 (1,820)	20 (200)	26 (260)	228 (2,280)
C2DE.A4C	221 (2,210)	25 (250)	30 (300)	276 (2,760)
C2DE.PSAX	223 (2,230)	24 (240)	29 (290)	276 (2,760)
Other	187 (1,870)	26 (260)	28 (280)	241 (2,410)
Total	1,937 (19,370)	237 (2,370)	262 (2,620)	2,436 (24,360)

For training, validation and testing datasets, clips are from separate echocardiographic videos. The numbers in parentheses indicate the number of images. 2DE, two-dimensional echocardiography; C2DE, contrast two-dimensional echocardiography; A2C, apical 2-chamber; A3C, apical 3-chamber; A4C, apical 4-chamber; PSAX, parasternal left ventricular short axis; Other, including parasternal left ventricular long axis, pulmonary artery long axis, and major artery short axis.

### View classification model architecture and training process

**FIGURE 1:**
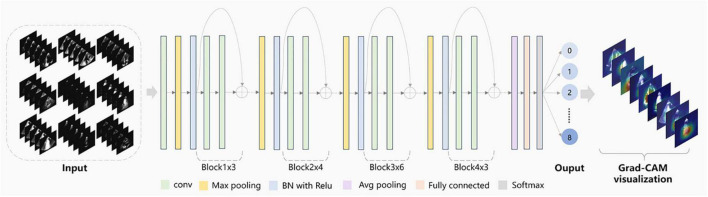
Schematic diagram of echocardiographic view classification.

View classification tasks were mainly performed by CNNs. This study chose the deep residual network with 34 layers (called EchoV-Net) to classify 9 classes of echocardiographic views. Numerous studies have demonstrated that the residual network (ResNet) can improve accuracy from considerably increased depth (19). EchoV-Net was developed based on ResNet, as shown in Table 3. We conducted comparative experiments to validate the performance. The detailed results are listed in Table 2 and Figure 1 of Supplementary materials. The overview diagram of view classification task is shown in Figure 1. For echocardiographic view classification, the model was trained to minimize the cross-entropy loss between the true label and prediction using an Adam optimizer with an initial learning rate of 0.001, and a batch size of 4 for 30 epochs. The learning rate was decayed by a factor of 0.9 each epoch. The 10 frames of each video were used as the model input. The final model was selected with the lowest loss of the validation dataset. The model was implemented on the software Python (version 3.7.10) and PyTorch (version 1.7.1), and on the server with one NVIDIA GeForce RTX3090 GPU and 24GB of memory.

**TABLE 3:** The architecture of EchoV-Net.

Layer name	Output size	Feature map
Conv1	128 × 128	5 × 5, 64, stride 2
Conv2	64 × 64	3 × 3 max pool, stride 2
		$\left[\begin{array}{cc} 3\times 3, & 64\\ 3\times 3, & 64 \end{array}\right]\times 3$
Conv3	32 × 32	$\left[\begin{array}{cc} 3\times 3, & 128\\ 3\times 3, & 128 \end{array}\right]\times 4$
Conv4	16 × 16	$\left[\begin{array}{cc} 3\times 3, & 256\\ 3\times 3, & 256 \end{array}\right]\times 6$
Conv5	8 × 8	$\left[\begin{array}{cc} 3\times 3, & 512\\ 3\times 3, & 512 \end{array}\right]\times 3$
	1 × 1	Average pool, 9-d fc, softmax

### Model evaluation and visualization

In the process of video classification, we evaluated 10 sampled images of each video, averaged the results, and assigned the video to the class with the maximum probability. The four metrics, namely accuracy, precision (also called “positive predictive value”), recall (also called “sensitivity”), specificity, and F1-score were used to evaluate the classification performance of EchoV-Net. All metrics were calculated separately in a single category (cardiac view), with the current category defined as a positive class, and the other 8 categories as negative classes. The overall accuracy is defined as the ratio of the number of correctly classified videos to the number of all samples. The top-1 accuracy is defined as the accuracy of the first prediction category that matched the true label. The top-2 accuracy is defined as the accuracy of the first two prediction categories that matched the true label. The precision is defined as the number of correctly classified positive samples divided by the number of true positive samples. The recall is defined as the number of correctly classified positive samples divided by the number of all positive samples. The specificity is defined as the correctly classified negative samples divided by all the negative samples. The F1-score is the harmonic average of precision and recall. Additionally, confusion matrices are calculated and plotted as heatmaps to visualize the results of multi-view classification.


$$A\,c\,c\,u\,r\,a\,c\,y=\frac{\text{TP}+\text{TN}}{T\,P+F\,P+T\,N+F\,N}$$

$$P\,r\,e\,c\,i\,s\,i\,o\,n=\frac{\text{TP}}{\text{TP}+\text{FP}}$$

$$R\,e\,c\,a\,l\,l=\frac{\text{TP}}{T\,P+F\,N}$$

$$S\,p\,e\,c\,i\,f\,i\,c\,i\,t\,y=\frac{\text{TN}}{T\,N+F\,P}$$

$$\mathrm{F1}-\mathrm{score}=2\times \frac{P\,r\,e\,c\,i\,s\,i\,o\,n\times R\,e\,c\,a\,l\,l}{P\,r\,e\,c\,i\,s\,i\,o\,n+R\,e\,c\,a\,l\,l}$$

The following strategies enhanced the interpretability of the classification model. The feature obtained by EchoV-Net was visualized using t-distributed stochastic neighbor embedding (t-SNE). t-SNE is a non-parametric dimensionality reduction technique that visualizes high-dimensional data by giving each sample a location in a two or three-dimensional map (20). In addition, gradient-weighted class activation mapping (Grad-CAM) was created to explain which critical anatomical structures (regions of the pixel) that affect image classification results (21).

### Re-evaluation by another expert

Due to fatigue and the similarity of views, there is inherent variation when observers explain echocardiograms, especially the apical views. Another expert specializing in cardiovascular imaging performed a blinded review of the samples, when there was disagreement between the initial label and the prediction by EchoV-Net. The expert received the paired echocardiograms and a set of labels including the initial human label and the prediction by EchoV-Net, and then tried to decide which label corresponded more closely to his or her evaluation of echocardiographic views. When reviewing the prediction results, experts recorded the reasons for misclassification of samples, such as poor image quality or contrast agent underfilling.

### Statistical analysis

All analyses were performed with R (version 4.1.2) or Python (version 3.7). The Kolmogorov-Smirnov test was used to assess the normality of patients’ age. Continuous variables were presented as the median (1st and 3rd interquartile range) and categorical variables were shown as frequency (%). The accuracy, precision, recall, specificity and F1-score were described as percentages. A value of *P* < 0.05 was considered statistically significant. The classification model was developed using the PyTorch package (22). DICOM images were processed by Pydicom and OpenCV 3.0.

## Results

**FIGURE 2:**
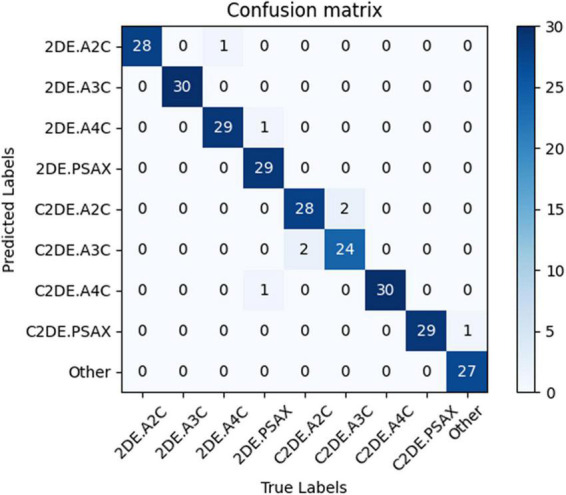
The confusion matrix demonstrated the results of view classifications within the test dataset. Numbers along the diagonal line represented successful classifications, while non-diagonal entries were misclassified. 2DE, two-dimensional echocardiography; C2DE, contrast two-dimensional echocardiography; A2C, apical 2-chamber; A3C, apical 3-chamber; A4C, apical 4-chamber; PSAX, parasternal left ventricular short axis; Other, including parasternal left ventricular long axis, pulmonary artery long axis, and major artery short axis.

**FIGURE 3:**
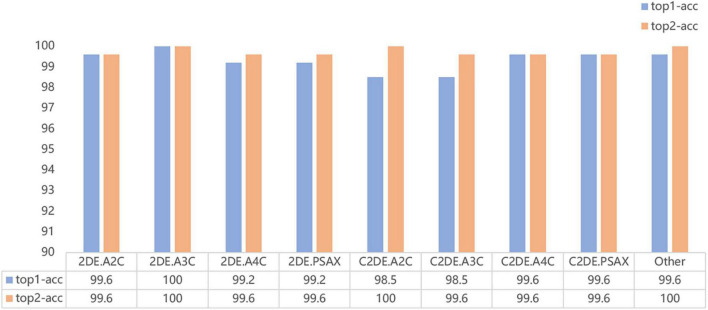
The accuracy of the classification model on the test dataset.

Our model successfully distinguished non-contrast and contrast echocardiographic views (as shown in the confusion matrix in Figure 2). The numbers on the diagonal are the number of correctly classified videos. On the test dataset, 96.9% (254 out of 262) of the videos are correctly classified. The top-1 accuracy of the model for the 2DE view is 99.5%, and it is 99.1% for the C2DE views (Figure 3).

The evaluation of the view classification is shown in Table 4. The A2C and A3C for 2DE and A4C and PSAX for C2DE are fully correctly classified. However, the recall of PSAX in 2DE and A2C and A3C in C2DE are reduced to 93.55, 93.33, and 92.31%, respectively.

**TABLE 4:** The results of view classification on the test dataset.

Cardiac view	Precision	Recall	Specificity	F1 score
2DE.A2C	96.55	100.00	99.60	98.25
2DE.A3C	100.00	100.00	100.00	100.00
2DE.A4C	96.67	96.67	99.60	96.67
2DE.PSAX	100.00	93.55	100.00	96.67
C2DE.A2C	93.33	93.33	99.10	93.33
C2DE.A3C	92.31	92.31	99.20	92.31
C2DE.A4C	96.77	100.00	99.60	98.36
C2DE.PSAX	96.67	100.00	99.60	98.31
Other	100.00	96.43	100.00	98.18

2DE, two-dimensional echocardiography; C2DE, contrast two-dimensional echocardiography; A2C, apical 2-chamber; A3C, apical 3-chamber; A4C, apical 4-chamber; PSAX, parasternal left ventricular short axis; Other, including parasternal left ventricular long axis, pulmonary artery long axis, and major artery short axis.

All metrics of the model are above 95% at the single video level. For the nine target views (i.e., A2C, A3C, A4C, PSAX and Other), the averages overall accuracy, top-2 accuracy, recall, precision, specificity, and F1 score are 97.0, 98.9, 96.9, 96.9, 100.0, and 96.9%, respectively.

**FIGURE 4:**
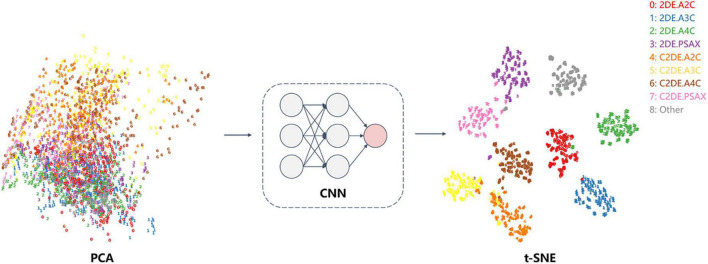
t-SNE visualization of view classification. On the left, each image was plotted in 2-dimensional space from 256 × 256 pixels by principal component analysis (PCA). The results showed that the data had no clear clustering pattern. On the right, the features of the fully connected layer of the CNN model (EchoV-Net) were projected to two-dimensional space by t-SNE, displaying that images were recognized into specific view categories.

**FIGURE 5:**
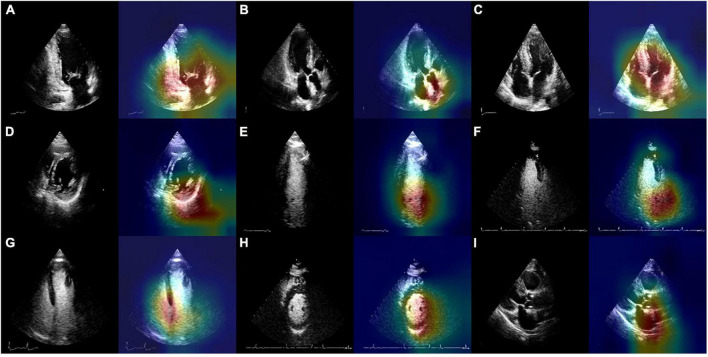
Original images and the results of EchoV-Net visualization of the most related regions for view recognition. **(A)** 2DE.A2C, **(B)** 2DE.A3C, **(C)** 2DE.A4C, **(D)** 2DE.PSAX, **(E)** C2DE.A2C, **(F)** C2DE.A3C, **(G)** C2DE.A4C, **(H)** C2DE.PSAX, and **(I)** Other. 2DE, two-dimensional echocardiography; C2DE, contrast two-dimensional echocardiography; A2C, apical 2-chamber; A3C, apical 3-chamber; A4C, apical 4-chamber; PSAX, parasternal left ventricular short axis; Other, including parasternal left ventricular long axis, pulmonary artery long axis, and major artery short axis.

The output of the fully connected layer in EchoV-Net is further interpreted by t-SNE (Figure 4) and Grad-CAM (Figure 5), showing obvious cluster results, and the classification criterion is consistent with the anatomical structure discerned by the cardiac sonographer. The Grad-CAM visual experiments indicate that the CNNs focus more on the mitral valve structure, the left ventricular outflow tract, and the cross of ventricle and atrium in A2C, A3C, and A4C, respectively. In addition, compared with non-contrast echocardiography, contrast echocardiography has a clearer cardiac contour, thus the regions of interest for CNNs focus more on the cardiac chamber cross junction (Supplementary materials and Figure 4). The F1 score of A4C in C2DE is 98.4%, and only 96.7% in 2DE (shown in Table 4).

**FIGURE 6:**
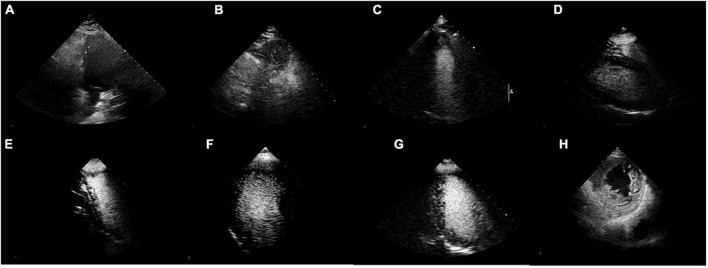
Misclassified samples. **(A–D)** Were of poor image quality. **(E–G)** Showed incomplete cardiac structures. **(H)** Was actually 2DE.PSAX, the top-1 prediction was C2DE.A4C, but the top-2 prediction was 2DE.PSAX.

Blinded reviews are performed for inconsistent results (shown in Table 5 and Figure 6). One expert with more than 5 years of clinical experience notes that 50% (4 out of 8) of the videos have considerable flaws and 37.5% (3 out of 8) of videos have incomplete cardiac structure, making it hard for the expert to identify the view. However, one video is misclassified by EchoV-Net despite good image quality. The expert prefers the initial human label in 87.5% (7 out of 8) cases based on the most likely outcomes (top-1 prediction). For one video, the expert prefers the prediction of EchoV-Net to the human label.

**TABLE 5:** Videos with the discordance between model prediction and human label.

Video	Human label	Top-1 prediction	Top-2 prediction	Expert results
565-14.dcm	2DE.A4C	2DE.A2C	2DE.PSAX	2DE.A4C
509-39.dcm	2DE.PSAX	2DE.A4C	C2DE.PSAX	2DE.PSAX
554-9.dcm	C2DE.A3C	C2DE.A2C	C2DE.A4C	C2DE.A3C
504-29.dcm	Other	C2DE.PSAX	Other	Other
558-47.dcm	C2DE.A2C	C2DE.A3C	C2DE.A2C	C2DE.A2C
306-29.dcm	C2DE.A3C	C2DE.A2C	C2DE.A3C	C2DE.A3C
172-53.dcm	C2DE.A2C	C2DE.A3C	C2DE.A2C	C2DE.A3C
314-62.dcm	2DE.PSAX	C2DE.A4C	2DE.PSAX	2DE.PSAX

## Discussion

Echocardiographic view classification is the basis of the analysis and interpretation of echocardiography. The above target views are the standard views recommended by the guidelines for clinical diagnosis. In this study, we proposed a CNN-based automatic echocardiographic view classification system, which classified the PSAX and A2C, A3C, and A4C views of contrast and non-contrast echocardiography. The system was developed by a training set of 688 cases containing 19,370 echocardiographic images. In the independent testing set of 83 cases with 2,620 images, the experimental results show that EchoV-Net accurately classified target views in contrast and non-contrast echocardiography, laying a foundation for subsequent AI-based cardiac function assessment and cardiovascular disease diagnoses.

Several factors lead to unsatisfactory view classification results. The intra-view variability of echocardiograms of the same cardiac view exists due to individual variations among subjects, different acquisition parameters (angle, depth, transducer performance, etc.), and the sonographer’s experience. The inter-view similarity of echocardiograms of different cardiac views exists due to similar information (e.g., valve and ventricular wall movement, left ventricle, etc.). The contrast agent fills the cavity of the left ventricle, enhancing visualization of endocardium boundaries, but it obscures the mitral valve, aortic valve and other structures, making it hard to which may the classifiers to distinguish A2C from A3C in C2DE. Besides, echocardiograms are mainly derived from positive cases, the abnormalities in cardiac anatomy increase the heterogeneity of the data. The speckle noise and clutter noise lower the clarity of the images, limiting the accuracy of view classification. For the PASX views in 2DE, the patient’s poor acoustic window makes it hard to identify the myocardium of the left ventricle. For the poor quality and low-level contrast images, it is necessary for experts to play an active role in quality control. What’s more, in the retrospectively collected data, view distribution is imbalanced. All the above-mentioned issues increase the difficulty in classifying the key echocardiographic views in contrast and non-contrast echocardiography.

The size and image quality of echocardiograms are essential for the development and validation of a model. In this study, we used a large dataset with a wide range of image quality that ensured data diversity and independence, making our model more robust to noise and poor image quality. However, in terms of computational efficiency, there is no comprehensive solution. A study carried out by Vaseli et al. attempted to overcome this problem. They adopted an knowledge distillation approach to compress the model and improve the efficiency of echocardiographic view classification (23). AI is often considered as a “black box,” which is challenging to understand, and thus we improved the interpretability of this model through several visualization methods, which showed that the model automatically classified echocardiograms depending on interpretable clinical features. The results indicate that the AI tasks have the potential to improve the work efficiency of sonographers and provide support for high-throughput analysis of echocardiography.

In future work, more echocardiographic views (e.g., parasternal long-axis views of the left ventricle, apical five-chamber view, etc.) need to be incorporated into our model, in addition to the frequently used ones. More high-quality and multimodal echocardiograms are expected to be used to improve the echocardiographic view classification system. In addition, we will continue exploring the causes of non-standard echocardiography to develop man-machine interactive quality control AI system and improve the performance of view classification.

## Conclusion

The main goal of the current study is to determine the feasibility and effectiveness of CNNs in view classification for contrast and non-contrast echocardiography. The results show that the EchoV-Net achieves expert-level view classification and accurately identifies the main categories in contrast-enhanced and non-contrast echocardiography. This study is expected to accelerate the automatic interpretation of contrast echocardiography and expand the clinical application of contrast echocardiography. In the future, the model also is expanded to classify other modalities of echocardiographic views (e.g., to distinguish colors, continuous-waves, pulsed-waves Doppler echocardiography), which has foundational significance for research, clinical practice, and sonographers’ training.

## Data availability statement

The original contributions presented in this study are included in the article/Supplementary material, further inquiries can be directed to the corresponding authors.

## Ethics statement

This study was approved by the Ethics Committee of Tongji Medical College, Huazhong University of Science and Technology, Wuhan, China. Written informed consent was not required for this study in accordance with the local legislation and institutional requirements.

## Author contributions

YEZ, JM, DN, MX, WX, and LZ conceived of the study. YIZ, SZ, and ML labeled the data. YEZ, JM, and ZSZ created and ran the data processing pipeline and wrote the manuscript. YEZ and JM designed and evaluated the deep learning models. CW, ZMZ, JC, XY, and LZ provided language help and proofed the manuscript. All authors contributed to the article and approved the submitted version.
