# Supplementary material for: Automatic view classification of contrast and non-contrast echocardiography
Source: Front Cardiovasc Med. 2022 Sep 14;9:989091. doi: 10.3389/fcvm.2022.989091 (PMC9515903; doi:10.3389/fcvm.2022.989091)
Supplement: Supplementary file 1 [file Data_Sheet_1.docx]

**Supplemental Table 1. Baseline characteristic.**

|  | Total  (N = 855) | Training  (N = 688) | Validation  (N = 84) | Testing  (N = 83) |
| --- | --- | --- | --- | --- |
| **Demographics** |  |  |  |  |
| Age (years) | 53(41, 62) | 53(40, 62) | 53(42, 62) | 51(43, 63) |
| Sex (male) | 601(70.3%) | 476(69.2%) | 65(77.4%) | 59(71.1%) |
| **History** |  |  |  |  |
| Myocardial hypertrophy | 360(42.0%) | 285(41.4%) | 37(44.0%) | 38(45.8%) |
| NCM | 85(9.9%) | 61(8.9%) | 11(13.1%) | 13(15.7%) |
| DCM | 17(2.0%) | 12(1.7%) | 3(3.6%) | 2(2.4%) |
| NCM & DCM | 32(4.0%) | 29(4.0%) | 2(2.4%) | 1(1.2%) |
| RWMA | 118(13.8%) | 98(14.2%) | 13(11.9%) | 7(8.4%) |
| others | 243(28.4%) | 203(29.5%) | 18(21.4%) | 22(26.5%) |
| **Years** |  |  |  |  |
| 2013 | 3 | 2 | 1 | 0 |
| 2014 | 6 | 6 | 0 | 0 |
| 2015 | 24 | 13 | 5 | 6 |
| 2016 | 42 | 34 | 3 | 5 |
| 2017 | 204 | 159 | 15 | 30 |
| 2018 | 250 | 211 | 29 | 10 |
| 2019 | 153 | 120 | 14 | 19 |
| 2020 | 63 | 58 | 3 | 2 |
| 2021 | 87 | 69 | 10 | 8 |
| other | 23 | 16 | 4 | 3 |
| **Equipment** |  |  |  |  |
| EPIQ 5 | 67 | 59 | 4 | 4 |
| EPIQ 7 | 1 | 1 | 0 | 0 |
| EPIQ 7C | 441 | 354 | 46 | 41 |
| EPIQ CVx | 7 | 6 | 1 | 0 |
| iE Elite | 106 | 79 | 9 | 18 |
| iE 33 | 196 | 160 | 20 | 16 |
| Vivid E9 | 22 | 17 | 3 | 2 |
| Vivid E95 | 2 | 2 | 0 | 0 |
| Vivid7 | 13 | 10 | 1 | 2 |

Data are expressed as median (interquartile range) or number (%). NCM = noncompaction of ventricular myocardium; DCM = dilated cardiomyopathy; RWMA = regional wall motion abnormality; Others = other conditions that required contrast echocardiography.

**Supplemental Table 2. The experimental results of different models on the validation dataset.**

|  | Accuracy | Mean Precision | Mean  Recall | Mean Specificity | Mean  F1 score |
| --- | --- | --- | --- | --- | --- |
| ResNet-18 | 94.9 | 95.5 | 94.7 | 99.0 | 94.9 |
| ResNet-34 | 95.8 | 95.8 | 95.9 | 99.0 | 95.8 |
| ResNet-50 | 95.8 | 95.8 | 96.0 | 99.0 | 95.7 |
| ResNet-101 | 93.7 | 94.3 | 94.0 | 99.0 | 94.0 |
| ResNeXt-50 | 96.2 | 96.3 | 96.5 | 100.0 | 96.3 |
| VGG-19 | 95.4 | 95.3 | 95.5 | 99.0 | 95.3 |
| Xception | 94.9 | 94.6 | 94.9 | 99.0 | 94.7 |
| EchoV-Net | **96.6** | **96.9** | **96.6** | **100.0** | **96.7** |

ResNet-34 is the original network. The kernel size of the first layer on EchoV-Net was changed from 7*7 to 5*5 based on ResNet-34. Except for VGG-19, the above models were trained using an Adam optimizer with an initial learning rate of 0.001, and a batch size of 4 for 30 epochs. The learning rate was decayed by a factor of 0.9 in each epoch. The original VGG-19 didn’t show good classification performance on the current dataset. We reduced the channel number of VGG-19 to improve the classification accuracy and used an Adam optimizer with an initial learning rate of 0.0001. The remaining hyperparameters remain unchanged.

**Supplemental Figure 1. Experimental results with different architectures on the validation dataset.**


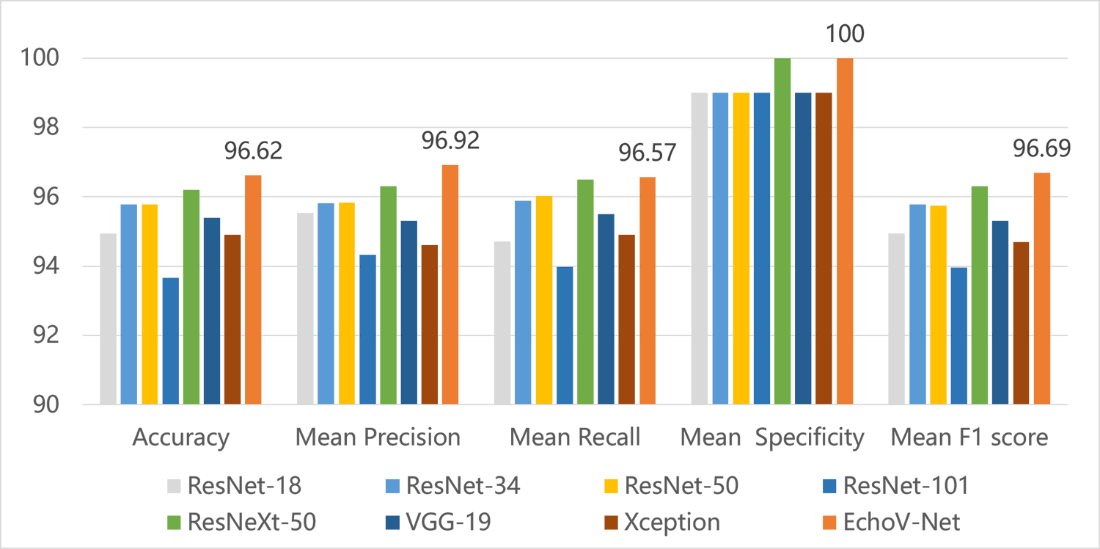


X in ResNet-X represents the number of convolutional layers. The kernel size of the first layer on EchoV-Net was changed from 7*7 to 5*5 based on ResNet-34. Except for VGG-19, the above models were trained using an Adam optimizer with an initial learning rate of 0.001, and a batch size of 4 for 30 epochs. The learning rate was decayed by a factor of 0.9 in each epoch. The original VGG-19 didn’t show good classification performance on the current dataset. We reduced the channel number of VGG-19 to improve the classification accuracy and used an Adam optimizer with an initial learning rate of 0.0001. The remaining hyperparameters remain unchanged.

**Supplemental Figure 2. Confusion matrix on the validation dataset.**


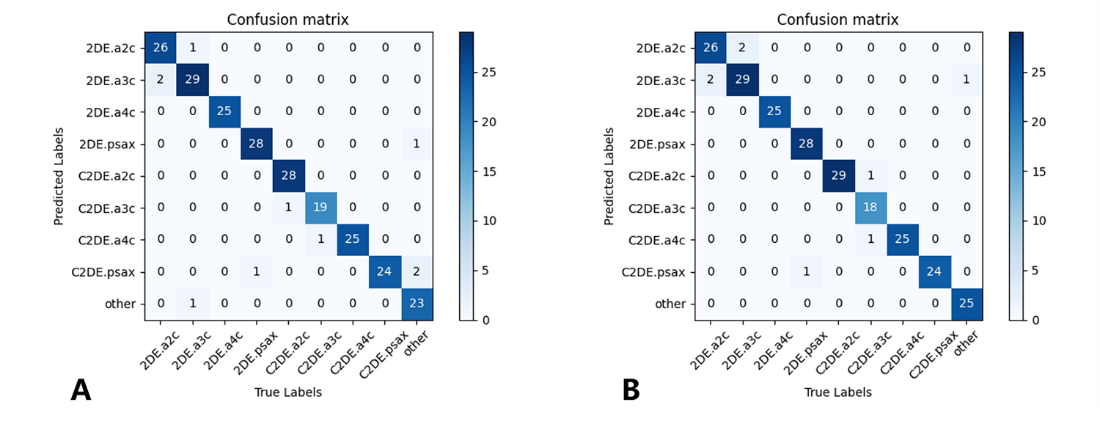


Numbers along the diagonal line represent successful classifications, while non-diagonal entries are misclassified. On the validation dataset, EchoV-Net (right) has fewer misclassified samples than ResNet-34 (left). **A**, confusion matrix of vanilla ResNet-34. **B**, confusion matrix of EchoV-Net.

**Supplemental Figure 3. Original images and visual classification results for misclassified samples on the test dataset.**


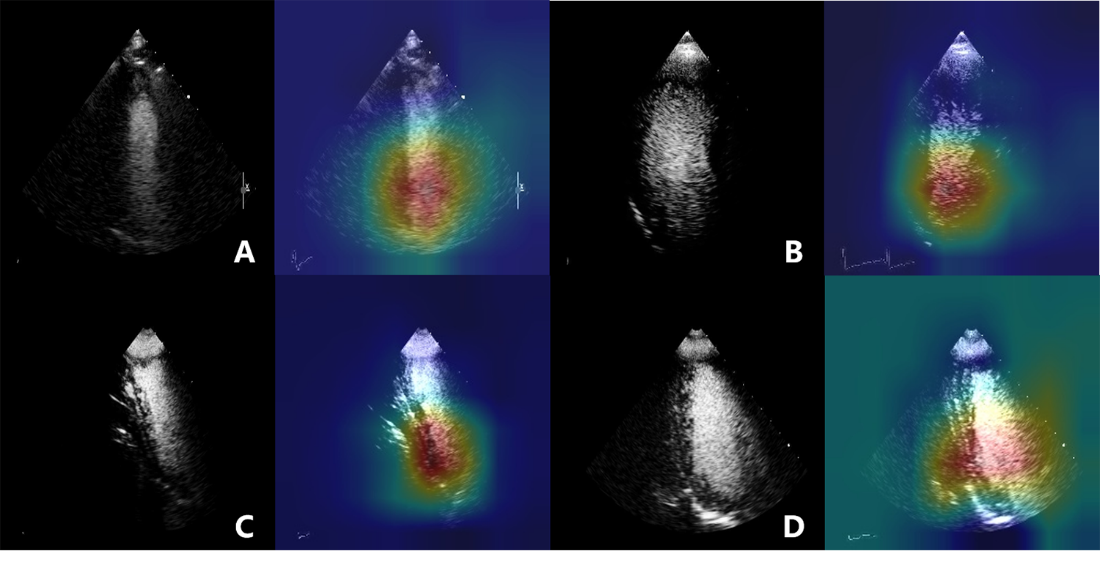


A and B, the A3C in C2DE misclassified into A2C. The model focused more on the mitral valve than on the left ventricular outflow tract. C and D, the A2C in C2DE misclassified into A3C. The model didn’t pay attention to the mitral valve.

**Supplemental Figure 4. Visual results based on Grad-CAM for correct classification samples on the test dataset.**


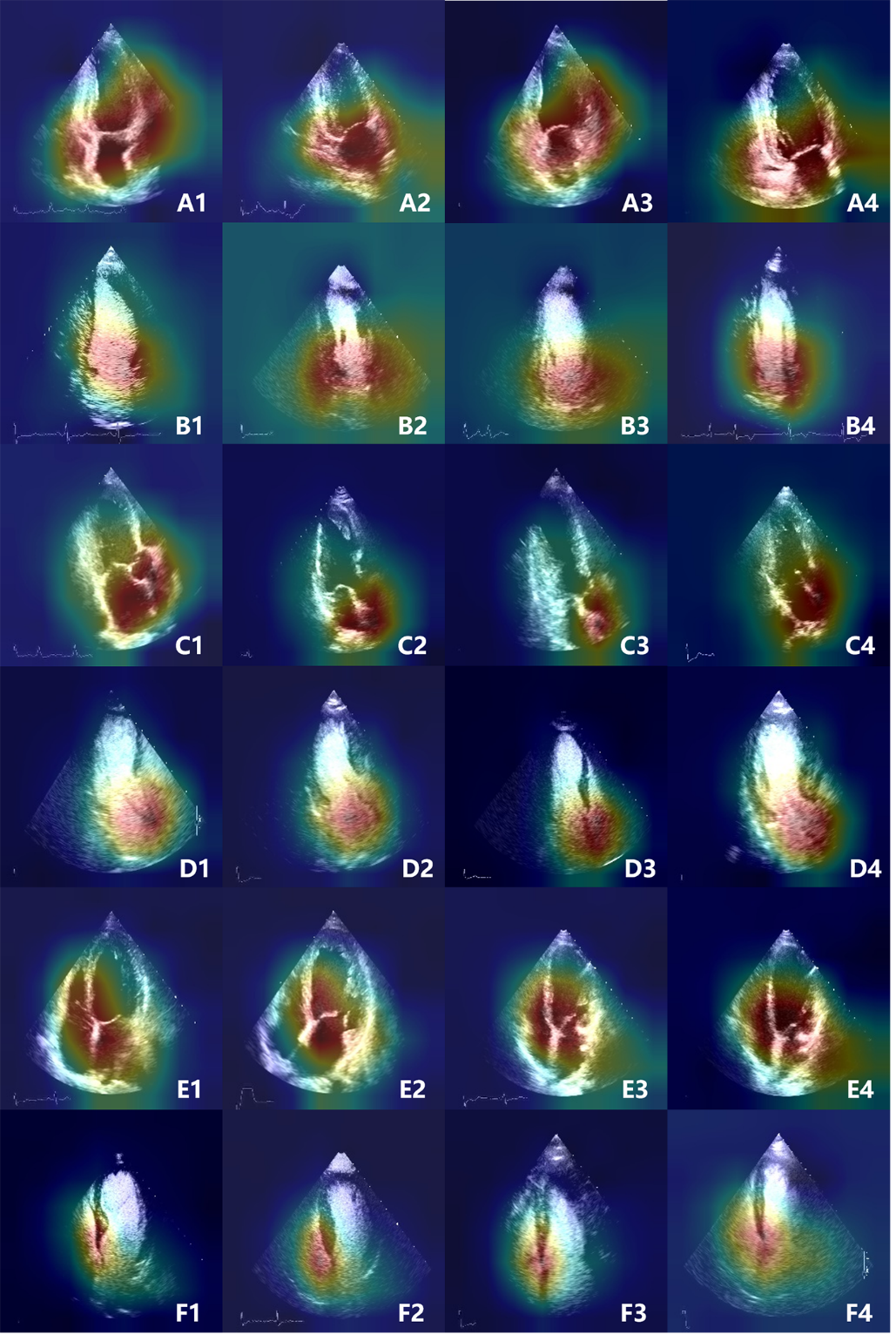


The visualization results of 24 echocardiography are shown. A, C, and E are A2C, A3C, and A4C in 2DE; B, D, and F are A2C, A3C, and A4C in C2DE. 2DE, two-dimensional echocardiography; C2DE, contrast two-dimensional echocardiography. The CNNs focus more on the mitral valve structure, the left ventricular outflow tract, and the cross of ventricle and atrium in A2C, A3C, and A4C, respectively.
